# Assessment of Alinity s Chagas^®^ as a Primary Diagnostic Test for Chronic Chagas Disease in a Non-Endemic Area of Europe (Barcelona, Spain)

**DOI:** 10.3390/life14101278

**Published:** 2024-10-08

**Authors:** Alba Abras, Cristina Ballart, Anna Fernández-Arévalo, Teresa Llovet, Montserrat Gállego, Carmen Muñoz

**Affiliations:** 1 Àrea de Genètica, Departament de Biologia, Universitat de Girona, 17003 Girona, Spain; 2 Secció de Parasitologia, Departament de Biologia, Sanitat i Medi Ambient, Facultat de Farmàcia i Ciències de l’Alimentació, Universitat de Barcelona, 08028 Barcelona, Spain; cristinaballartferrer@ub.edu (C.B.); mgallego@ub.edu (M.G.); 3 Institut de Salut Global de Barcelona (ISGlobal), Hospital Clínic, Universitat de Barcelona, 08036 Barcelona, Spain; 4 Servei de Microbiologia, Hospital de la Santa Creu i Sant Pau, 08025 Barcelona, Spain; tllovet@santpau.cat (T.L.); cmunoz@santpau.cat (C.M.); 5 Institut de Recerca Biomèdica Sant Pau, 08041 Barcelona, Spain; 6 CIBERINFEC (Centro de Investigación Biomédica en Red de Enfermedades Infecciosas), Instituto de Salud Carlos III, 28029 Madrid, Spain; 7 Departament de Genètica i Microbiologia, Universitat Autònoma de Barcelona, 08193 Barcelona, Spain

**Keywords:** Alinity s Chagas, Architect Chagas, chronic Chagas disease, Europe, serology, Spain

## Abstract

Chagas disease (CD) has become a worldwide problem due to globalization. In Europe, most cases are imported and are diagnosed in the chronic phase by two serological tests, as recommended by the World Health Organization. Chemiluminescent microparticle immunoassays (CMIAs) are an emerging alternative to the diagnostic standard. We aimed to validate the CMIA Alinity s Chagas^®^ as a primary diagnostic test for chronic CD following its replacement of Architect Chagas^®^, with an amended signal-to-cut-off (S/CO) ratio of ≥6. Laboratory results and clinical data were collected retrospectively from 774 sera from an at-risk population tested for CD in Barcelona during 2020–2022. Negative results required no further testing, and those with a S/CO ratio ≥ 0.8 were confirmed by a second serological assay, according to the common practice. Four per cent of the samples (31/774) were determined to be seropositive by Alinity s, 93.5% of which (29/31) had an S/CO ratio ≥ 6. Almost all the samples could be directly classified by the corrected S/CO. Alinity s Chagas^®^ was validated as a single test for chronic CD diagnosis by raising the S/CO to ≥6. Its implementation could provide faster results and help reduce CD underdiagnosis in non-endemic countries.

## 1. Introduction

Chagas disease (CD), caused by *Trypanosoma cruzi*, is a leading health problem in Latin America, where it affects 6–8 million people [1]. Traditionally confined to rural Latin America and restricted to vector-borne transmission, increased human mobility has spread CD to urban centers in endemic countries, as well as to non-endemic countries, transforming it into a global health concern [2].

The disease occurs in two consecutives stages named the acute and chronic forms. The acute phase generally evolves asymptomatically or with mild and nonspecific symptoms such as persistent fever, fatigue, arthralgia, myalgia, and lymphadenopathy [3]. Vectorial transmission by hematophagous triatomine bugs (Hemiptera: Reduviidae) may result in so-called portal of entry signs at the site of inoculation of *T. cruzi* parasites into the human body: unilateral palpebral edema (Romaña sign) or indurated cutaneous lesion (chagoma) [3,4]. Oral outbreaks related to the consumption of *T. cruzi*-contaminated food or beverages usually have a more severe symptomology [5]. Parasitemia is usually high during the acute stage, so the diagnosis of *T. cruzi* infection at this point is based on a microscopic observation of parasites in the blood by parasitological methods and/or detection of their DNA by means of molecular techniques like PCR [6]. In untreated immunocompetent individuals, the acute form usually resolves spontaneously within 4–8 weeks and progresses to a chronic phase [7]. Most cases settle in the indeterminate stage, which is asymptomatic and may persist throughout the patient’s life. However, one to three decades after the acute infection, around 30–40% of chronically infected patients will develop a chronic symptomatic CD with severe cardiac and/or gastrointestinal involvement, mainly cardiomyopathy, megaesophagus, and megacolon [3]. During the chronic phase, low and intermittent parasitemia compromises a direct diagnosis. Therefore, CD confirmation relies on the detection of IgG antibodies against *T. cruzi* by serological testing [6].

In Europe, where most cases are imported, the diagnosis of chronic CD is particularly important due to the presence of 4.6 million Latin American migrants, 4.2% of whom are estimated to be in this phase of the infection [8,9]. For this migrant population, it is essential to be diagnosed and treated before CD progresses to cardiac and/or digestive disorders [10]. In addition, in the absence of the vector, autochthonous cases can occur through blood transfusion, organ transplant, and from mother to child (congenital transmission) [2]. Considering that the two first routes of transmission are well controlled in most countries and that migration flows from Latin America to Europe have a young age profile (average age of 37.6 years) and show a gender asymmetry towards females (55.3% of Latin American migrants), women of a child-bearing age are the major contributors to new cases of CD in non-endemic areas, resulting in increased congenital infection [9,11,12]. However, despite all these facts, there is a clear lack of policy regulations for CD screening in people who are at epidemiological risk [10,12]. This, together with the lack of CD awareness, leads to an underestimation of cases that hides the real situation of the disease in Europe and hinders access to treatment [13,14].

Eight years ago, our research team, led by Dr. M. Gállego (Universitat de Barcelona and ISGlobal) and Dr. C. Muñoz (Hospital de la Santa Creu i Sant Pau, HSCSP, of Barcelona and Universitat Autònoma de Barcelona), proposed an algorithm to improve chronic CD diagnosis, in which the chemiluminescent microparticle immunoassay (CMIA) Architect Chagas^®^ (Abbott Laboratories, Wiesbaden, Germany) was used as a single test, with the signal-to-cut-off (S/CO) ratio increased from 1 (as recommended by the manufacturer) to 6 [15]. After its implementation in HSCSP of Barcelona, Spain, the effectiveness of the modified protocol was assessed in 2021. It was concluded that CMIA Architect Chagas with the amended S/CO ratio was a suitable and cost-effective single test for CD diagnosis in non-endemic countries [16]. Following its replacement of Architect Chagas in August 2020, the aim of the present study was to validate Alinity s Chagas^®^, processed on the new Alinity s System^®^ (Abbott Laboratories, Wiesbaden, Germany), as a primary test for chronic CD diagnosis, applying an S/CO ratio ≥ 6.

## 2. Materials and Methods

In this study, data from all serum samples received at the HSCSP laboratory with a request for CD testing during the period from August 2020 to December 2022 were included in the analysis. Following this inclusion criterion, the sera of 774 individuals were selected, and their laboratory results and clinical information were retrospectively collected. The serological testing request was based on the following assumptions: (i) individuals with epidemiological antecedents: (e.g., pregnant women from CD-endemic areas, women of a child-bearing age from CD-endemic areas); (ii) individuals with medical disturbances potentially related to chronic CD (e.g., alterations in the electrocardiogram, difficulty in swallowing and/or defecation); (iii) individuals with a family history of CD; and (iv) diagnosed chronic CD patients. Laboratory results were obtained using the computer system OpenLab, and clinical data were compiled trough the software Systems, Applications and Products for data Processing (SAP) (Logo 7.60). All samples were anonymized before being included in the study. The patient cohort consisted of 656 women and 118 men, aged 10–89 years, with an overall mean age of 37 years (median = 34; IQR = 11).

As established in the routine procedure for the diagnosis of CD in the HSCSP, all samples were tested by the fully automated assay CMIA Alinity s Chagas. The test was performed according to the manufacturer’s instructions with an assayed sample volume of 98 μL and a dead volume of 200 μL. The duration of the test was 37 min per serum, and the cost per determination was EUR 6.48. The chemiluminescent reaction was measured in relative light units (RLUs). Due to the excellent sensitivity previously reported for CMIA [17,18], which was later reaffirmed by subsequent studies [15,19,20], in 2017, the HSCSP implemented CMIA as the primary test for routine CD diagnosis. Therefore, a negative result (ratios of sample RLUs/cutoff value [S/CO] <0.8) was considered conclusive as negative. Gray-zone (S/CO from ≥0.8 to <1) and positive results (S/CO from ≥1) were confirmed by the ELISA test BioELISA Chagas (Werfen, Lliçà d’Amunt, Spain) until May 2021. The test was performed manually according to the manufacturer’s instructions, with all the necessary reagents supplied with the kit. The test time was about 2 h and the cost per sample, according to data from the period when the technique was still in use at HSCSP, was EUR 6.00. Results were interpreted following the kit indications: ratios of sample absorbance/cutoff value (Abs/CO) of <0.9 were considered negative, ≥1 positive, and the gray zone was from ≥0.9 to <1.

**Figure 1:**
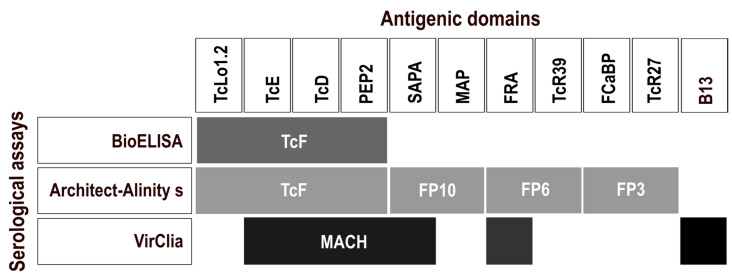
Recombinant antigens with the antigenic domains used in the Architect Chagas and Alinity s Chagas assays (Abbott Laboratories, Wiesbaden, Germany) in comparison with BioELISA (Werfen, Lliçà d’Amunt, Spain) and VirClia (Vircell, Granada, Spain). Dark gray boxes represent the antigenic domains in BioELISA, light gray boxes the antigenic domains in Architect Chagas and Alinity s Chagas, and black boxes the antigenic domains in VirClia. Most antigenic domains are included in multi-domain recombinant antigens, which are indicated within the boxes for each serological assay (TcF, FP10, FP6, FP3, and MACH).

As of June 2021, the chemiluminescent immunoassay (CLIA) Chagas VirClia (Vircell, Granada, Spain) was implemented as the confirmatory technique of choice for CD diagnosis in the HSCSP, replacing the BioELISA Chagas test. Hence, samples received in the laboratory from this date onwards with a CMIA Alinity s result of ≥0.8 were retested by VirClia instead of BioELISA. VirClia was also performed according to the manufacturer’s instructions using the automatic processor. The sample volume analyzed was 5 μL, with a dead volume of 150 μL. The test duration was 1 h and 21 min and the cost per determination was EUR 5.60. Results were interpreted as recommended in the kit: ratios of S/CO < 0.9 were considered negative, >1.1 positive, with a gray zone from ≥0.9 to ≤1.1. The final interpretation was based on coincident results in both techniques (primary and confirmatory), and in case of discordance, the laboratory requested an additional sample to be analyzed using the same serological flowchart. The recombinant antigens included in each test are summarized in Figure 1.

## 3. Results

**Figure 2:**
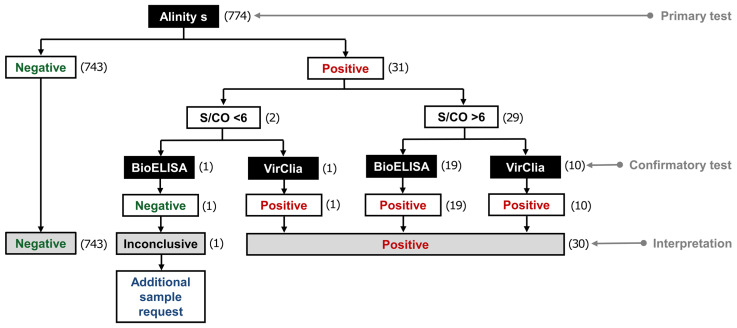
Serological results obtained in the 774 samples included in the study. The number of samples is bracketed.

Of the 774 serum samples analyzed, 743 (96%) tested negative based on Alinity s, with their S/CO values ranging from 0.01 to 0.74 (Figure 2). The remaining 31 samples (4%) tested positive; 29 (93.5%) had S/CO ratios > 6 (between 7.41 and 14.72), and 2 (6.5%) had S/CO values < 6 (5.28 and 5.41) (Table 1).

Among these two samples, one was confirmed as positive by the VirClia retest, whereas the other gave discordant serological results, being positive based on Alinity s (S/CO < 6) and negative based on BioELISA (patient 14 in Table 1). In the latter, the additional sample required also obtained discordant results between the two same serological assays (positive based on Alinity s with a S/CO of 5.01 and negative based on BioELISA with an Abs/CO of 0.65). Chagas disease was confirmed based on the laboratory results from OpenLab and clinical and epidemiological data from the SAP system.

A total of 16 samples included in the study corresponded to patients with a previous diagnosis of chronic CD and with multiple serological test records in the OpenLab computer system of the HSCSP. Of these, 11 were negative in all samples tested, and the remaining 5 were positive in all samples recorded. The latest included additional samples from patients 13, 14, 18, 19, and 25 presented in Table 1. In all cases, the request for CD testing was related to treatment follow-up, except in the case of patient 14, which is detailed in the discussion section.

## 4. Discussion

In Europe, as in other non-endemic countries, most people with CD are in the chronic phase, since they have acquired the infection in their country of origin [11]. Early diagnosis and treatment of these patients avoid the clinical evolution of CD and its associated cost to the healthcare system [10]. On the other hand, women of a child-bearing age, with a relevant role in new cases of autochthonous CD, represent the LA migrant group who are most affected by underdiagnosis [9,12,21]. Therefore, the establishment of a chronic CD surveillance system that includes serological screening and treatment programs for the population at risk would help prevent new infections and control the disease, both in imported and autochthonous cases.

CMIA-based techniques have demonstrated high sensitivity in diagnosing CD, which is especially important in the chronic phase [22]. Although their cost per determination is in line with that of the other assays used in the present study (around EUR 6.00), CMIAs require large-scale equipment, hampering their use in CD-endemic countries beyond large urban areas or for screening in blood banks [16]. This has probably been one of the reasons why the Pan American Health Organization (PAHO) still recommends the classic diagnostic gold standard to confirm infection in patients with suspected chronic CD [23]. However, these constraints are not applicable in non-endemic countries, where an efficient, cost-effective, and easy-to-use system may alleviate the high rates of CD underdiagnosis (70–90%) [13,14,21]. Another point to keep in mind is that the PAHO, as an international public health agency, needs clear evidence supported by numerous diagnostic test evaluation studies before including new techniques in its official recommendations [24]. In this sense, the present study contributes to the knowledge of the diagnostic performance of CMIA, adding to previously published works in this regard [15,16,17,18,19,20,25,26,27]. In earlier studies, we demonstrated that Architect Chagas with a higher S/CO ratio of ≥6 was a promising single test for chronic CD diagnosis [15,16], with only a few samples needing confirmation by a second serological assay. Architect Chagas has now been replaced by Alinity s Chagas, processed on the new Alinity s System, but shares the same principle: paramagnetic microparticles coated with four *T. cruzi* recombinant antigens and chemiluminescence as the detection system [28] (Figure 1). The present work aimed to validate the new Alinity s assay, maintaining the modified S/CO ratio.

Among the 774 samples tested by Alinity s, 31 were positive (4%), and only 2 samples, with S/CO ratios < 6, required additional testing when applying the higher S/CO. Discordant results between Alinity s and BioELISA were only found in one individual, in which the retest in a new sample also gave discordant results (patient 14 in Table 1). In this case, CD was confirmed after a clinical data review. This corresponds to patient A1 in Abras et al. [16], a Paraguayan woman with untreated chronic CD with a history of four discordant serologies between Architect (positive with S/CO 5.35 to 6.65) and BioELISA (negative) in 2016–2017 and *Leishmania* sp. infection ruled out by the *Leishmania*-Spot IF (BioMérieux, Marcy l’Etoile, France). The discordant results are attributable to the higher sensitivity of Architect-Alinity s, which includes more *T. cruzi* antigens than BioELISA (TcF) [28,29] (Figure 1). Regarding VirClia, the test also includes a significant proportion of recombinant antigens, but all of them are shared with Alinity s except for the trypomastigote surface protein B13 [30,31] (Figure 1). In comparison, Alinity s has a higher number of antigenic domains or peptide epitopes per test, which may increase its sensitivity. The four hybrid recombinant proteins represent the three morphological forms of *T. cruzi* (i.e., trypomastigote, epimastigote, and amastigote) and contain antigenic regions that are recognized by antibodies that are present in individuals with acute and chronic CD [17,32]. The broad antigenic coverage of the assay, coupled with the incorporation of highly conserved antigenic proteins of *T. cruzi* including tandemly repeated amino acid sequence motifs, has the potential to encompass the genetic diversity of *T. cruzi* (i.e., discrete typing units, DTUs) [15,17]. This may be of particular interest to avoid possible erroneous results depending on the circulating genotype(s) of the parasite in different geographical regions and may provide a solution to one of the main drawbacks of serological testing for CD, such as discordant serological results that are attributable to antigenic differences among *T. cruzi* DTUs [33,34]. However, more studies are needed involving representative samples of the different genotypes of *T. cruzi* (i.e., TcI to TcVI and TcBat), as well as the wide geographic distribution of the parasite in the Americas. In fact, the use of recombinant proteins, and particularly multiepitope proteins, applied to the immunodiagnosis of CD has been pointed out as promising due to their high diagnostic performance and accuracy [35].

On the other hand, although not represented in the present study, the high sensitivity of Alinity s for detecting *T. cruzi* can lead to nonspecific reactions or cross-reactions, resulting in false positive (FP) results. However, they can be avoided by the proposed adjustment of the S/CO ratio to ≥6. This is corroborated by the results obtained in the previous evaluation of Architect Chagas performed by our group, in which all FP sera (6/315) achieved values of <5 S/CO (between 1.8 and 4.6) [15]. In that study, moreover, practically all the FP sera (5/6) were from *Leishmania*-infected patients. In this regard, the current work has two main limitations. Firstly, the samples could not be tested in parallel by Architect and Alinity s due to insufficient sera for reanalysis. Nevertheless, as both tests share the same principle and antigens, similar results can be expected. Secondly, samples from patients with other infections were not included to evaluate cross-reactions, which would be mandatory to reduce the S/CO ratio below 6, as indicated elsewhere [19]. However, considering that only 0.3% of the sera exhibited an Alinity s S/CO ratio between ≥0.8 and <6, further S/CO modification would not impact the diagnostic costs.

## 5. Conclusions

CMIA can be used as a single test to confirm *T. cruzi* infection in patients with suspected chronic CD in non-endemic areas by raising the S/CO recommended by the manufacturer. The diagnostic value of Alinity s Chagas was validated, as was the applicability of the amended S/CO of ≥6. According to our results, over 99% of samples could be directly diagnosed by this strategy without needing further analysis. Thus, integrating CD diagnosis in the high-throughput automated Alinity s System, which simultaneously diagnoses different infections on a single device, can facilitate testing and provide faster results, ultimately helping to reduce the levels of CD underdiagnosis in non-endemic countries.

## Figures and Tables

**Table 1:** Serum samples positive in Alinity s (*n* = 31) with their respective confirmatory test results (BioELISA or VirClia).

Patient	Year	Country of Origin	Sex (F/M)	Age (years)	Alinity s (S/CO)	BioELISA (Abs/CO)	VirClia (S/CO)
1	2020	-	F	35	8.14	2.20	-
2	2020	-	F	51	8.20	4.72	-
3	2020	Bolivia	F	34	8.40	2.80	-
4	2020	-	F	49	8.48	3.49	-
5	2020	-	F	55	8.75	7.34	-
6	2020	-	F	58	9.75	4.61	-
7	2020	-	F	52	9.77	12.24	-
8	2020	-	F	43	10.24	9.70	-
9	2020	Bolivia	F	51	10.89	6.76	-
10	2020	-	H	37	10.89	5.50	-
11	2020	-	F	38	11.55	6.40	-
12	2020	-	F	66	13.09	2.86	-
13	2021	Spain ^1^	M	12	5.28	-	1.03
14	2021	Paraguay	F	35	5.41	0.20	-
15	2021	Bolivia	F	32	7.41	-	2.06
16	2021	Bolivia	F	43	8.08	8.96	-
17	2021	Bolivia	F	35	8.16	6.02	-
18	2021	Spain ^1^	M	13	8.71	3.47	-
19	2021	Bolivia	F	58	8.99	-	6.22
20	2021	Bolivia	F	49	9.19	-	2.99
21	2021	-	F	34	9.59	8.37	-
22	2021	-	F	51	9.67	2.76	-
23	2021	Bolivia	M	55	10.62	-	2.09
24	2021	-	F	56	10.63	7.75	-
25	2021	Bolivia	F	48	12.69	-	6.95
26	2021	-	F	61	14.72	1.48	-
27	2022	Bolivia	F	24	7.82	-	3.09
28	2022	Bolivia	M	42	9.65	-	2.69
29	2022	Bolivia	F	40	11.88	-	3.09
30	2022	Bolivia	F	36	12.72	-	3.01
31	2022	Argentina	F	39	13.94	-	4.79

F, female; M, male; S/CO, signal-to-cut-off ratio; Abs/CO, sample ratio of absorbance/cut-off value. ^1^ Born to a mother with CD.

## Data Availability

The data underlying this study cannot be made publicly available, as the protocol by the Ethics Committee of the HSCSP explicitly indicated that patients’ data would be anonymized and confidentially preserved. Public availability would compromise patient privacy and breach compliance with the protocol approved. Researchers who need to access the data may request the information from the corresponding author.
